# Cultural continuity, traditional Indigenous language, and diabetes in Alberta First Nations: a mixed methods study

**DOI:** 10.1186/s12939-014-0092-4

**Published:** 2014-10-19

**Authors:** Richard T Oster, Angela Grier, Rick Lightning, Maria J Mayan, Ellen L Toth

**Affiliations:** Department of Medicine, University of Alberta, 4100 Research Transition Facility, 8308 114 Street, Edmonton, AB T6G 2V2 Canada; Piikani Blackfoot Nation, Box 3273, Brocket, AB T0K 0H0 Canada; Ermineskin Cree Nation, Box 418, Maskwacis, AB T0C 1N0 Canada; Faculty of Extension, University of Alberta, 2281 Enterprise Square, 10230 Jasper Avenue, Edmonton, AB T5J 4P6 Canada

**Keywords:** Indigenous population, Diabetes mellitus, North America, Language, Qualitative research, Cross sectional analysis

## Abstract

**Introduction:**

We used an exploratory sequential mixed methods approach to study the association between cultural continuity, self-determination, and diabetes prevalence in First Nations in Alberta, Canada.

**Methods:**

We conducted a qualitative description where we interviewed 10 Cree and Blackfoot leaders (members of Chief and Council) from across the province to understand cultural continuity, self-determination, and their relationship to health and diabetes, in the Alberta First Nations context. Based on the qualitative findings, we then conducted a cross-sectional analysis using provincial administrative data and publically available data for 31 First Nations communities to quantitatively examine any relationship between cultural continuity and diabetes prevalence.

**Results:**

Cultural continuity, or “being who we are”, is foundational to health in successful First Nations. Self-determination, or “being a self-sufficient Nation”, stems from cultural continuity and is seriously compromised in today’s Alberta Cree and Blackfoot Nations. Unfortunately, First Nations are in a continuous struggle with government policy. The intergenerational effects of colonization continue to impact the culture, which undermines the sense of self-determination, and contributes to diabetes and ill health. Crude diabetes prevalence varied dramatically among First Nations with values as low as 1.2% and as high as 18.3%. Those First Nations that appeared to have more cultural continuity (measured by traditional Indigenous language knowledge) had significantly lower diabetes prevalence after adjustment for socio-economic factors (p =0.007).

**Conclusions:**

First Nations that have been better able to preserve their culture may be relatively protected from diabetes.

## Introduction

The term cultural continuity was described by Kirmayer et al. [1] as “culture as something that is potentially enduring or continuously linked through processes of historical transformation with an identifiable past of tradition”. Over 15 years ago Chandler and Lalonde [2] published their now salient and influential work on First Nations youth suicide and cultural continuity (defined as the contemporary preservation of traditional culture). As the only empirical evidence, their work has been used often to contend the importance of traditional Indigenous culture in protecting Canadian Indigenous people from the many contemporary health crises they face. Through use of government mortality data on First Nations communities, it was found that youth suicide incidence rates varied markedly in British Columbia First Nations [2,3]. Using publically available data, the authors then selected ‘proxy measures’ to classify these First Nations based on the extent to which they had sustained or attained cultural continuity. They found an inverse relationship between those First Nations with greater sustained cultural continuity and youth suicide rates [2,3].

A closer look at the proxy measures Chandler and Lalonde [2,3] utilized suggests that the authors may not have been measuring solely cultural continuity, but also local administrative autonomy or self-determination [4]. In 2007, Chandler and Lalonde updated their data to include Aboriginal language use as a marker of cultural continuity, finding First Nations with greater than 50% of members having Indigenous language knowledge having youth suicide rates six times less than those First Nations with less than 50% of members having Indigenous language knowledge [5].

Despite the influence of Chandler and Lalonde [1,2], to our knowledge their work has not been repeated in their own or other contexts, or in relation to other major Indigenous health problems, such as type 2 diabetes. Our intent with the current study was to do just that. Type 2 diabetes and its complications burden Indigenous populations in Canada at rates two to five times higher than that of the general population [6,7].

### Research approach and ethics

First, we believed we needed a better understanding of what cultural continuity and self-determination meant in the Alberta First Nations context, whether they are related, and their association with health and diabetes. To do this, we used an exploratory sequential mixed method research design partitioned into two phases which started with and emphasized a qualitative description of a phenomenon, building to a second smaller quantitative piece that was informed by the qualitative findings [8]. For the qualitative phase, we interviewed Cree and Blackfoot leaders living in and working for their Nations. For the subsequent quantitative phase, we conducted a cross-sectional analysis of administrative data and publically available data.

A key aspect to our study was the linking of the qualitative and quantitative phases. We followed the process described by Creswell and Plano Clark [8] for connecting data during the data collection stage. Briefly, the findings of the qualitative phase shaped the research question and data collection strategy for the quantitative phase [8]. Due to the exploratory nature of the research, we placed precedence on the rich knowledge we gained from the qualitative participants and examined public data in attempts to find quantitative evidence to enhance/supplement the qualitative findings.

We acquired ethical approval from the Human Research Ethics Board, University of Alberta, which adheres to the Tri-Council Policy Statement - 2 guidelines for Research Involving the Aboriginal, Inuit and Métis Peoples of Canada [9]. After reviewing and discussing the study information letter, each participant in the qualitative phase provided written informed consent. We used numerical codes as identifiers and the participants remained unidentifiable throughout the study. The anonymity of the communities in the quantitative phase was also a priority, and was achieved by using numerical codes and by limiting community-specific details. We worked with an advisory group of Indigenous individuals brought together regularly to advise on projects involving Indigenous health. The group’s role was to assist in interpretation of data, dissemination of the findings, and to ensure the entire study was conducted in a culturally appropriate manner.

## Qualitative phase

### Methods

To delve into cultural continuity, self-determination, and their relationship with health from the perspective of First Nations leaders, we used qualitative description. This approach is applied when a descriptive summary of a phenomenon is needed [10], and is one of the most commonly utilized approaches in qualitative research related to health [11]. Qualitative description stays close to the words of participants, produces rich and straight descriptions with ‘low-interference’ data interpretation, and is often used for mixed method inquiries for knowledge building and subsequent study using quantitative methods [10,11].

#### Recruitment and sample

The first author (RTO), with some assistance from a research assistant, recruited participants through personal contacts and through telephone calls and e-mails to current Band Council members as listed on the Aboriginal Affairs and Northern Development Canada (AANDC) website. To ensure province-wide representation, we strived to include participants from each of the three Treaty areas (Treaty 6, Treaty 7, and Treaty 8). We purposefully sampled 10 adult (20 years or older) First Nations participants that were currently, or had recently been (within three years), an elected community official (Band Councilor or Chief) in a First Nation within Alberta.

Of the 10 participants, four were from Treaty 6, two were from Treaty 7 and four were from Treaty 8. Eight of the participants were Cree and two were Blackfoot. Seven of the participants were males, whereas three were females. The majority of participants (seven) were currently working as members of Chief and Council for their respective Nations at the time of their interview, whereas three were previously on Band Council.

#### Data generation

RTO, again with some assistance from a research assistant, carried out one-on-one semi-structured interviews with participants. Participants were provided with an interview schedule prior to the interview so they could familiarize themselves with some of the questions to be discussed. The interviews were conducted in English, and were audio-recorded and transcribed verbatim. Participants were prompted to discuss what community cultural continuity and self-determination meant for them and if/how these concepts related to health and diabetes. Examples of prompting questions included: What does community-level self-determination mean to you? How do you feel self-determination and health are related (if at all) in your community? What does cultural continuity mean to you? How do you feel cultural continuity and health are related (if at all) in your community? How do you feel self-determination and cultural continuity are related (if at all)? Interviews were conversational and participants were encouraged to add any other information they felt was important.

#### Data analysis

RTO analyzed the data using a qualitative content analysis process [12] that took place concurrently with data collection. We used Atlas.ti qualitative computer software for data management. For coding, RTO read and re-read the interview transcripts, to highlight and recognize persistent concepts and underlying patterns. Highlighted sections were grouped in categories in separate files. The individual files were similarly read and re-read, and the categories were then described thoroughly and draft findings were written. The categories were also considered together to identify any common threads. At this stage, the draft findings were then discussed in a series of meetings that included RTO, MJM, ELT, and some members of our research group. During these meetings the categories were further refined and defined via collaborative discussion. It was also decided at this stage, since it was agreed that further interviews were not likely to reveal new information or insight, that data saturation had been reached and data collection should be concluded. As a final step, we shared and discussed the findings with the qualitative participants that had been interviewed (those that were interested in doing so) to enhance the trustworthiness and accuracy of the data, as well as to provide an occasion for further empowerment for the participants. This was done via email and in-person meetings (depending on the wishes of each participant), and each participant was provided their transcript for review as well as an overview of the findings. The findings were verified by the participants as an accurate portrayal of their collective experiences. Participants also had the opportunity to be further involved in data analysis and dissemination.

#### Rigor

We achieved strength and rigor during the qualitative research process by adhering to the strategies for qualitative description set out by Whittemore, Chase, and Mandle [13] and Milne and Oberle [14] of authenticity, credibility, criticality and integrity. Briefly, authenticity refers to paying close attention to the voices of participants, and was attained by purposeful sampling, participant-driven data collection and accurate transcription. Credibility entails capturing and depicting a truly insider perspective, and was attained through researcher reflection on the believability of the results. Criticality, or being questioning of one’s work, was attained through reflection on the critical appraisal of decisions made throughout the research process. Integrity pertains to honesty and probity and was attained through peer review, participant follow-up, and reflection on researcher bias. RTO kept a reflective personal journal throughout the entire research process to allow for reflection and critical appraisal, with particular reflexivity paid to the role of being a non-Indigenous researcher interpreting the voices of Indigenous leaders.

### Findings

#### Conceptualizing cultural continuity

The importance of traditional culture, or “being who we are”, was the most widespread and recurring theme participants mentioned, and the most relevant to health and diabetes. The need for a strong attachment to and respect for culture was emphasized time and again as the basis of any thriving and healthy First Nation, as one participant said, “All things flow from our culture.” Participants described their culture as “sacred”, “essence”, “well-being”, “livelihood”, “balance”, “respect”, “way of life” and “everything”. Culture contains all of the teachings and direction on “how to walk in this world” and includes, but is not limited to, traditions, values, knowledge, hunting and trapping, living off the land, traditional food, medicines, games, sweats, spirituality, ceremonies, celebrations, praying, and language. Participants viewed having culture permeate all aspects of life as “an Indigenous way to live” and “a harmonious way to live”.

Participants described traditional language in particular as a crucial and inseparable piece of culture. The participants felt when First Nations “live from their language” they are “maintaining all that (they) believe in and all that you’ve been born from”. The participants believed traditional culture and language to be one and the same. Without use of their traditional language, Nations were deemed incapable of succeeding since language is at the center of culture and provides the blueprint for how to live and survive:

Elders always speak of the importance of our language. Who we are is determined through our language. We speak our language and that determines where you come from, what your culture is, and even how we used to go with the different seasons in terms of following those traditional paths. Regardless of where you go, if you have that language our culture is in there… So once you lose that, what do you have left? Because our beliefs come from that in terms of how we govern ourselves. It comes in terms of how we eat, and in terms of how we educate ourselves and conduct ourselves in that full circle.

The participants felt that traditional culture, and having their culture continue into future generations, is inextricably linked to their health. During the interviews many health problems faced by First Nations were discussed such as diabetes, cancer, heart disease, oral health problems, sexually transmitted diseases, addictions and alcoholism, and mental health issues. The participants consistently related these problems back to culture. The loss of culture often was seen as the root cause, and re-connecting with traditional culture was seen as the solution in many instances:

Everybody has been gifted with the ‘how’ (knowledge), to deal with themselves and we have to realize that. We have to get back to that. Every Nation in this country and in this world has been gifted with that ability. Even the animals know how to heal themselves… Indian people were like that. They healed themselves, but times have changed.

Some participants referenced the Medicine Wheel and a “holistic view of health that includes mind, body, spirit, emotions” to illustrate how culture and health influence each other. A connection to culture is believed to impact and “bring balance” to each of the four corners of the Medicine Wheel. For diabetes specifically, the disease was described as “a cultural thing” and some participants explained more direct positive effects of increased physical activity and consumption of traditional foods, and consequently less western foods, resulting from cultural activities such as hunting, fishing, trapping, and “living off the land”.

Participants explained that having a First Nation that is firmly attached to its traditional culture and language is the foundation of a collective identity, which contributes to health and well-being. Some of the participants explained how a First Nation cannot endure and “know where it is going” without first understanding “who we are and where we come from”. Identity in Nations stems from culture, as one participant explained “it’s all laid out for us as to who we are and what we should be. Rituals, traditions, and the culture, that’s what moulds you and will guide you in your life”. A collective identity and “just being who we are” was also believed to be vital and concurrent with a sense of belonging. One participant talked about the universal need of belonging and how it is linked to culture:

Everybody has to have a sense of belonging. Everybody has to feel like they’re part of something. And if you don’t, where are you going to end up? Probably on the street and homeless because you think nobody cares. And that’s not just Aboriginal people, that’s all people. They NEED to have a sense of belonging… You have a sense of pride because you have a sense of belonging. That is the value in our culture and in an individualistic world you don’t have that.

A secure sense of culture leads to intact and healthy First Nations, and Nations with integrity, as one participant expressed “the success is how solid they are as a community in their faith system, you know, their culture. Right now we’re hurting. So we got to go back, and we got to be solid”. The participants believed culture strengthens the integrity and well-being of Nations by making possible capable and ethical leadership, a sense of peace and tranquility through spirituality, well-adjusted families and healthy relationships, and strong support systems:

Our traditional knowledge services have been such a shining light in the darkness because it’s so promising, and people are starting to have a lot of respect for it and wanting to be a part of it. The values generated out of that will take care of people, whom will take care of each other. The compassion, the generosity, those are our traditional values. When we remain a collective society, and when that’s our value system, that takes a lot of work and (leads to) ethics in leadership, respectful relationships, spirituality and healthy relationships.

According to the participants, Nations are in a state of cultural rehabilitation as they struggle to maintain cultural continuity: “(Our people) are lost. They don’t know their language. They don’t know where they come from, where they are going”. Some spoke of a desperate need to listen to Elders who are urging Nations and their members “to go back our traditional ways, to our language, to our spirituality, to our rituals and traditions, and our culture”. Much of this cultural rehabilitation seems to hinge on connecting the youth to traditional culture: “our young people today they need to know who they are, who their ancestors are, where they come from, and who their relatives are”.

#### Conceptualizing self-determination

How self-determination at the First Nation level was understood can be summarized by the statement of one participant: “being a self-sufficient Nation”. For all of the participants, having self-determination meant a Nation that has independence, freedom, and collective control over their destiny. Living in a self-sufficient Nation was seen as means of survival and way of life prior to colonization and the signing of Treaties. Some of the participants spoke of “the past’s self-determination” where individuals “remained a collective society” and where “tribes and clans of families could travel without having to worry, to hunt, to follow the seasons, and to have a life of freedom”. Participants described an idyllic autonomous state that culminates in healthy Nations that are able to rely on their own members and local government, and “not depend on anybody”. One participant echoed this response:

Self-determination means self-sustaining. You have to be able to sustain a community. You have to feed them, you have to water them, and you have to house them locally. You have to have those efforts centralized locally, just like any other community.

The autonomy and ensuing self-sufficiency of the past has been lost and denied as Nations were forced to transition from a “nomadic life of freedom” that naturally led to “health and wellness”, to Nations that are controlled by and dependent on the dominant, conquering, and colonizing society’s government. One participant remarked “the older generation seemed more self-determined than the new generation, ‘cause this generation seems more dependant”. Participants went on to describe how ‘the ideal’ self-determination is afforded to other non-Indigenous municipalities in Alberta, and perhaps Indigenous communities in other provinces, but is something First Nations in Alberta have little prospect of. One participant described this controlling situation as being “stuck in a corner or backed up against the wall” whereas another felt First Nations are “sabotaged no matter what”. Participants repeatedly linked this lack of self-determination to poor health outcomes, including diabetes, particularly since the participants felt the federal government that has usurped control over First Nations “can never really understand the health issues we have because they’ve not grown up with those issues”.

Although immediate and complete self-determination was deemed unrealistic, there was a recurrent sentiment of hope to one day regain the self-sufficiency and subsequent healthfulness of the past, as one participant stated “it is definitely a goal, to be not just self-determined but self-reliant”. Another remarked that “dependency is one of the things that’s been created, and that’s something we’ve been trying to get away from”. Self-determination was viewed as an inherent right that is crucial in improving the health and social inequities that exist for Indigenous people, and part of “honoring the Treaties”. Nations are in a continuous struggle for self-determination where “you got to keep working at it every day”.

Some participants spoke of other factors that might contribute to self-determination in First Nations such as economic development, increased employment, increased number of educated people, increased local autonomy, reclaiming traditional lands, and engaging the mainstream society with reciprocity in mind. However, the participants frequently explained that self-determination in Nations is unimportant and irrelevant unless it is rooted in traditional culture and “ways of being”: “self-determination it can be miscued I think if a lot of people don’t speak the language because our Elders say that’s who we are. It’s enmeshed in our language”. Another participant went on to clarify:

Self sustaining is tied right back to our culture, our source of life. It’s tied right back to the environment, and it’s tied right back to living within our means and living respectfully, and not being arrogant about the lives we live and taking more than we need. That’s a sustainable community because it’s built on a sacred foundation, it’s built with respect, and the respect is our primary virtue of who we are. Respect each other, respect this land, respect the spiritual elements that are out there, and a relationship to all of that. We need to carry that through to everything that we do.

#### Barriers to cultural continuity and self-determination

The participants felt their Nations were fighting a seemingly endless battle to secure their culture as a means of bettering or rescuing Nations and gaining self-determination. Although the participants were not specifically questioned about barriers, they were discussed at great length and became a prevailing theme throughout the interviews. These barriers, because of the strong links between cultural continuity and health, were believed to instigate and perpetuate poor health outcomes.

One of the most debilitating and recurrent barriers was the lasting impact of historical traumas experienced by Indigenous people and Nations. The participants depicted how colonial policies of assimilation that aimed to “kill the Indian” have wreaked havoc on Nations through not only the loss of culture and well-being, but the loss of lives, land, autonomy, values, integrity, dignity, and way of life. One participant reflected on the ongoing effects of such policies: “my people have been so colonized a lot of them don’t want to be Indians”. The residential school system was one such colonial policy that continues to impact Nations and their members:

It is another issue that to me has affected self-determination. I do see a lot of issues that have occurred from residential school and how that’s carried out through generations today. It’s a generational issue and the problems created from residential schools are not going to be changed over night. That pain will transfer through to the youth.

One participant described residential schools as “one of the most fierce modern-day forms of genocide”. The negative impacts of residential school are profound and ongoing, resulting in “diabetes”, “broken communities”, “loss of parenting skills”, “addictions, suicides, and marital breakups”, “apprehended children”, “lifeline (culture) severed”, “shame”, loss of “a voice”, “mental health problems”, “contaminated families”, “disarray and chaos”, and “pain”. Residential schools were believed by the participants to have nearly destroyed their culture. Every participant had their own personal family story of residential schools and its impacts. One such story was so poignant we felt it was necessary to include it in full:

Residential schools were concentration camps. People don’t want us to make reference to the Nazis and of the concentration camps, but they were here. They were real. A kid is brought in… Shave your head… You’re given a number. You’re given a uniform… You’re four years old, you’re put in an institution, and you’re institutionalized for fourteen years of your life. They did that to us. My grandpa seen his brother thrown in a hole. He was dead. They made the children bury their own. It was a mass grave. They made these little guys bury their brothers, and tell ‘em, “ok, get back to the fields and work”. So they taught these children how to have no emotion, no love, no family, not knowing how to be family members. The federal government doesn’t want to recognize the issues of the day scholars (students that attended residential schools that did not require them to stay overnight), but the day scholars were just as abused as the residential school people were abused at night, only the day scholars were abused during the day… There was an Elder that once said “the stories will come out of the residential schools through the floors and the walls”. He said that in Cree. At the time it didn’t make sense. When they started ripping down the residential schools, they were finding fetuses buried in the floors, in the walls. They were finding skeleton bones in the incinerators of fetuses. So GIRLS were being raped and having babies, but they were being aborted and murdered. So you can imagine how severe the mental issues that arose. I guess one of my conclusions that came from that is anybody that went to these residential schools came out of there with some form of mental health issues. The alcoholism and drug abuse is one of the (results) that you see. Look at the anger that’s out there, and it’s brought out by alcoholism, the domestic violence, the violence in our communities, and the suicides. Those are offshoots of residential school. They WERE normal when they came in, they weren’t normal when they left. How can we have self-determination when we all have mental issues?

Another significant barrier the participants pointed out was controlling, disrespecting and disempowering government policies. One participant described these policies as “a sickness of colonization we fight every day” and “a colonial government based on bloodshed, and land and money”. The participants felt the Indian Act especially denied their Nations’ self-determination: “everything is dictated to us by the Indian Act. Our whole life is run by the government”. Some of the participants described how the Indian Act disallows Indigenous people from borrowing or having collateral which puts extreme limits on getting a mortgage, starting a business, or economic development. Some participants thought that the Indian Act and other government policies have created dependency in First Nations that “is a killer”. When speaking about the possibility of self-government one participant remarked:

We can’t call ourselves self-governing if we have to depend on another government to sustain us. But yet that other government isn’t letting us take full control of our natural resources in our traditional lands and territories. If we have a society that is too dependant on the government you are going to breed poverty, and poverty also breeds dependency.

Many of the participants believed the federal government does not provide enough funding to First Nations such that they are under-funded compared to provincial municipalities, and that this underfunding impacts their health. One participant compared this to the underfunding for youth in school: “I know for an example that in a nearby city students are getting like $16,000 or maybe $14,700 per child while we get only $7,000, maybe”. The participants felt that the provincial and federal governments are continuing to act unethically and take advantage of First Nations. One participant felt the government treats Indigenous people as second rate citizens: “they are only Indians; we can cut ‘em back, who cares”. Another participant explained how First Nations in Alberta are being short-changed by the government regarding natural resources:

I always use that metaphor of, I picture the Crown sitting around a big buffet table and they just have this plethora of food and you know just catered to the nines, and the scraps that fall off their table they’re like “oh okay, go give that to the First Nations community”. We’re expected to function on shrinking budgets… There’s no revenue sharing agreement. So that means that the federal government or the provincial government gets to have all the money, all the say, and all the development of natural resources. And natural resources are what makes the world go around and what makes the economy. You can only get so far in selling moccasins worldwide.

In many instances participants viewed western society and its values as a barrier to cultural continuity and self-determination. Some participants explained how western society continues to encroach on and influence First Nations, and that the erosion of Indigenous languages was a direct result of this “outside influence”. One participant described how traditional culture is increasingly limited, and sedentary behaviours consequently heightened, due to this western influence:

There’s so much at play with the dominant society because you have TV, you got media, and people are constantly in the midst of the dominant society. We’ve been so inundated with the dominant society. Our communities have become disrupted. Our medicines have been destroyed. Here in our community we’re surrounded by towns, and villages, and farming people. We don’t have the capacity to go in the bush. We don’t have water to go fishing, to go hunting and gathering. All of that has been taken away because we’re closed in. We’re like a jail here.

Some participants thought much of the non-Indigenous population is uninformed of the true history of Indigenous people in Canada, including the intergenerational impact of residential schools, the government’s “lack of honoring the Treaties”, and the dependency and discrimination of the Indian Act. Some believed this lack of awareness leads to racism and intolerance towards Indigenous people that has become so systemic that “it has become normal”. Participants described racism and discrimination having a severe deflating effect on Nations where some individuals are ashamed of their own culture.

#### Summary

Taken together, the participants told a collective story. Traditional culture and language were described as one and the same, and the blueprint for survival and health in First Nations. Self-determination on the other hand was conceptualized as a state of self-sufficiency that was once a way of life but is now denied and currently unrealistic for Alberta First Nations. Nations are fighting to rehabilitate their culture and consequent self-determination, with ensuing improved health and well-being in mind, in the face of government policy, the intergenerational effects of colonization, and ever-mounting western influence.

## Quantitative phase

The qualitative findings were used as the basis for designing the second phase of our mixed methods approach. It was clear from the qualitative participants that cultural continuity is of paramount importance for healthy and enduring First Nations, and cultural continuity is directly and indirectly linked to health, well-being, and diabetes. We wanted to examine whether quantitative data would support this. Since self-determination was viewed as unachievable in Alberta and something that can only exist in the future if it stems from cultural continuity, we felt that measuring differences in cultural continuity between Nations would be appropriate whereas measuring differences in self-determination was not likely to bear fruit. Therefore, in the second phase of our mixed methods approach, we aimed to determine whether diabetes prevalence rates in First Nations in Alberta were related to cultural continuity. It was our intention to supplement the qualitative finding that cultural continuity is inextricably linked to health by conducting this quantitative analysis.

### Methods

We used a cross-sectional retrospective design. First Nations adult (20 years and older) crude diabetes prevalence rates for all Alberta communities for the year 2005 were available from the administrative databases of Alberta Health. Analysts from Alberta Health applied the National Diabetes Surveillance System algorithm to identify diabetes cases, which requires an individual to have either two physician visits within two years or one hospitalization for diabetes (International Classification of Diseases - 9 codes 250 or International Classification of Diseases - 10 codes E10-E14) [15]. The algorithm excludes pregnant women who are assumed to have gestational diabetes. First Nations residents were identified by Alberta Health from the Alberta Health Care Insurance Plan Registry file and defined as any Alberta resident registered under the Indian Act of Canada and entitled to Treaty status with the Canadian Government.

First Nations crude prevalence rates for 585 Alberta communities were obtained from Alberta Health. The advisory group assisted in identifying 31 of the 585 communities as First Nations that we could collect publically available data for. For each community, First Nations individuals constituted more than 80% of the population.

We identified AANDC as the only publically available source of First Nation-specific data. AANDC is a federal department responsible for supporting Indigenous communities and has on their website (http://www.aadnc-aandc.gc.ca/eng) statistics on community demographics, education, workforce, income, and language that is derived primarily from national censuses and required reporting. The most recent available data from AANDC is for the year 2011, which we utilized for this analysis. For each of the 31 Nations we were able to determine Indigenous language knowledge rates, which we used as a measure of cultural continuity. This is in line with our qualitative findings which revealed the importance of traditional culture and its relevance to health and diabetes, as well as the unyielding connection between culture and traditional language. It must be noted that in our quantitative analysis we use traditional language as a proxy for cultural continuity. We recognize that traditional language and cultural continuity are not equivalents; rather traditional language is just one component of the concept of cultural continuity. However, we were limited by the data that was available via AANDC. We were able to gather data on median household income, unemployment rates, and high school completion rates to control for socio-economic differences.

Once the dataset was prepared, community names were removed. All analyses were conducted using STATA statistical software. Descriptive statistics on diabetes prevalence were conducted. Simple and multiple regression analyses were performed to evaluate the relationship between diabetes prevalence and the independent variables.

### Findings

**Figure 1:**
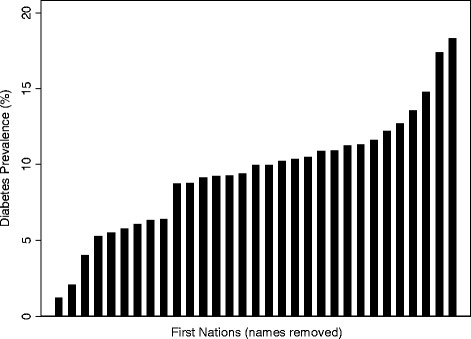
**Crude diabetes prevalence by First Nations community in Alberta for the year 2005.**

**Figure 2:**
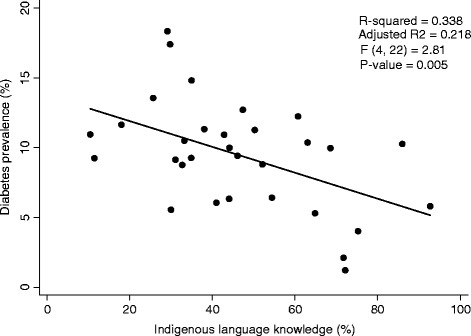
**Crude diabetes prevalence by Aboriginal language knowledge for the year 2005.** P-value reflects multiple linear regression test result (adjusted for socio-economic factors).

The overall mean crude diabetes prevalence for the identified 31 First Nations was 9.5%. Nevertheless there were striking differences among these Nations with diabetes prevalence as low as 1.2% and as high as 18.3% (Figure 1). Similarly, Indigenous language knowledge prevalence varied markedly between Nations with a low of 10.5% and a high of 92.8%. Table 1 shows the average values and simple linear regression results (as a predictor of diabetes) for median household income, unemployment rate, high school completion rate, and Indigenous language knowledge rate. Only Indigenous language knowledge was a significant predictor of diabetes in simple linear regression. This relationship remained in multiple regression after adjustment for socio-economic factors, β = −0.973, t = −2.96, p =0.007 (Figure 2).

**Table 1:** **Average values across all 31 First Nations and simple linear regression results (as predictors of diabetes prevalence) of independent variables**

	**Average (range)**	**Simple linear regression β coefficient**	**P-value**
Median household income	$32,092	-.0001079	0.313
	($20,765 - $49,729)		
Unemployment rate	25.6%	.1421939	0.235
	(14.3% - 39.4%)		
High school completion rate	11.6%	-.1213925	0.437
	(4.7% - 22.2%)		
Indigenous language knowledge rate	46.4%	-.0926571	0.005
	(10.5% - 92.8%)

## Discussion

Our findings transcend much of the generalizing and deficit-focused Indigenous health literature. Our qualitative findings suggest that cultural continuity or “being who we are”, which is intricately linked with traditional language, is fundamental to health in Alberta First Nations. Our quantitative results support this, and suggest that some First Nations in Alberta have been better able to preserve their culture (as measured by Indigenous language knowledge) and consequently are relatively protected from diabetes.

Overall trends indicate that Canada’s Indigenous populations, First Nations especially, are excessively burdened by type 2 diabetes and its complications [6]. The most recent age-adjusted data for Alberta shows 13.5% of adult First Nations people have diabetes compared to only 6.0% of adults of the general population [7]. Epidemiological literature in this area has led to mostly generalized results, with scarce data on community differences. We show that generalizing obscures the differences in prevalence that exist among different First Nations. Some Nations experience diabetes prevalence rates far exceeding the provincial average whereas others experience very little diabetes. A similar “saw tooth” pattern was also observed for youth suicide rates in British Columbia by Chandler and Lalonde [2,3]. Although a few other studies show diabetes prevalence may be lower among remote First Nations [16-18], reasons for this protection have been speculative. We have shown the first empirical evidence (to our knowledge) that First Nations differences in diabetes prevalence are due in-part to differences in cultural continuity.

Our findings substantiate qualitative work suggesting the key to improving Indigenous health and lessening the burden of type 2 diabetes is the contemporary revitalization and continuation of the culture. In her seminal work with Anishinaabe First Nations adults with diabetes in Manitoba, Garro [19] showed that the health care message of individual responsibility for diabetes is short-sighted and that diabetes is likely a symptom of colonization. Follow-up work with Opaskwayak First Nations individuals in Manitoba pointed to a loss of autonomy and cultural vulnerability specifically [20]. Traditional holistic healing was identified as a primary coping method for dealing with the stress and trauma associated with living with diabetes among Indigenous individuals in Winnipeg, Manitoba [21]. Indigenous adults with diabetes living in the Bella Coola Valley of British Columbia reported the importance of traditional foods and medicines, as well as ceremonial practices, in their diabetes experience, and specified a need for health care professionals to understand Indigenous culture [22]. Recently, urban First Nations individuals from Eastern Ontario proposed that cultural destruction and colonization have led to low self-esteem and self-worth and eventual diabetes in First Nations people [23].

Our findings also build on the work done by Chandler and Lalonde [2,3,5], which suggests that cultural continuity contributes to a sense of a shared and persistent identity which in turn limits suicide as an option in the chaotic lives of many First Nations youth. Our findings also suggest that cultural continuity is protective (in this case against diabetes). We were able to show that this relationship is significant even after adjusting for socio-economic differences between Nations, which Chandler and Lalonde [2,3,5] were unable to account for in their original reports on suicide. The debilitating social circumstances that many Indigenous Nations endure are no secret and were alluded to by our qualitative participants. They uniformly depicted how cultural continuity works to stabilize Nations in these turbulent times, not solely through preserving their shared identity, but also by enhancing social support, encouraging healthy interpersonal relationships, supplying spiritual tranquillity, and ultimately providing tangible strategies for living and surviving in the world. Participants also linked cultural continuity to increased physical activity and consumption of traditional foods resulting from hunting, trapping, and fishing.

The work of Chandler and Lalonde [2,3] has also been used as evidence of a link between self-determination and health in First Nations, at least in British Columbia. More recently, increasing local autonomy over health services was found to be associated with decreasing rates of hospitalization for ambulatory care sensitive conditions in Manitoba First Nations [24]. Also, all-cause mortality, cardiovascular mortality and hospitalisation with cardiovascular disease were shown to be significantly lower among Aborigines living in ‘decentralized’ communities (with greater community control) compared to other Australian Aborigines [25].

Measuring differences in self-determination between Nations, and any relationship with diabetes prevalence, was not explored in our quantitative phase as our qualitative findings suggest that self-determination is extremely limited in Alberta First Nations. This lack of self-determination in Alberta is apparent when looking at the percentage of First Nations that have signed Health Services Transfer Agreements with the federal government. In 1999 only 7% of eligible Alberta First Nations had done so, which was the lowest of any province in Canada [26]. The national average of eligible First Nations that had signed these agreements was 41% in 1999, with rates as high as 70% in Saskatchewan [26].

There are limitations to our study. We interviewed formal leaders in local government, but we did not capture the perspectives of informal/traditional leaders (such as Elders). There may be contradictory opinions, particularly in respect to ways to move forward, but the unanimity of the responses with respect to the meanings of Indigenous culture is reassuring. It should be made clear that the Treaties First Nations are subject to in Alberta do not exist in British Columbia where the work of Chandler and Lalonde [2,3] was conducted. Caution is needed when interpreting the cross-sectional results, which were from 2005. It is also important to reiterate that our measure of cultural continuity, traditional language, is a proxy measure. Future work should aim to quantify other components of cultural continuity in relation to diabetes and health. We are unable to distinguish between type 1 and type 2 diabetes in our administrative data, however we know that type 2 diabetes constitutes the great majority of cases, and is the most influenced by the social determinants of health. Although we were able to control for socio-economic factors in our quantitative analyses, the contribution of other potential contextual predictors to the regression model such as age, lifestyle, overweight/obesity, and social/lived environment, etc. could not be assessed.

## Conclusions

Much of the literature related to ethnicity and health has shown clear health disparities between Indigenous and non-Indigenous populations, often with diabetes at the forefront [27,28]. Our findings go beyond, and suggest that one of the key underlying determinants of these disparities is cultural continuity. We suggest that interventions aimed at reducing type 2 diabetes rates of First Nations people should work to break down the barriers to cultural continuity and continue the recent revitalization of First Nations cultural reclamation spurred by the Royal Commission on Aboriginal Peoples [29]. To do so, researchers, health care providers, and policy makers need to collaborate with, understand, and engage individual Nations rather than generalizing policies and approaches. Traditional Indigenous culture, including (but not limited to) traditional language, requires urgent protection and revival. For cultural rehabilitation to continue the responsibility also lies with governments and the non-Indigenous population of Canada to permit autonomy, to work towards decolonization and true reconciliation, and put an end to the marginalization of Indigenous people.
